# Effect of antenatal depression on maternal dietary intake and neonatal outcome: a prospective cohort

**DOI:** 10.1186/s12937-016-0184-7

**Published:** 2016-07-11

**Authors:** Ayesha Saeed, Tahira Raana, Amina Muhammad Saeed, Ayesha Humayun

**Affiliations:** 1 Home Economics (Food and Nutrition), College of Home Economics, Gulberg, Lahore, Pakistan; 2 Department of Nutrition Sciences, Faculty of Health Sciences, University of South Asia, 47- Tufail Road, Lahore, Pakistan; 3 Department of Human Development and Family Studies, Govt. College of Home Economics, Gulberg, Lahore, Pakistan; 4 Kinnaird College for Women University, Lahore, Pakistan; 5 Department of Public Health and Community Medicine & Department of Undergraduate Medical Education, SheikhKhalifa Bin Zayed Al-Nahyan Medical College, Lahore, Pakistan; 6 Registrar Shaikh Zayed Medical Complex, Lahore, Pakistan

**Keywords:** Depression, Diet, Fetal Growth Retardation, Gestational age, Apgar score, Low Birth Weight, Macronutrients

## Abstract

**Background:**

In Pakistan, incidence of antenatal depression ranges from 18 to 80 %, which goes undiagnosed, resulting in maternal and neonatal implications. The current study aimed to examine the association of antenatal depression with maternal dietary intake and neonatal outcome.

**Methods:**

A hospital-based, prospective cohort study was conducted on 94 middle class antenatal attendees coming to a tertiary care hospital in Lahore, Pakistan at the beginning of second trimester. Participants fulfilling eligibility were enrolled consecutively after taking written informed consent. Exposure group was identified by Edinburgh Postnatal Depression Scale (EPDS) and cohort members were followed till after delivery. Maternal dietary intake was assessed by 24-h Recall and Food Frequency Checklist, while neonatal outcome was identified through patient files before discharge. Data on potential confounders was collected. Loss to follow up was 13 % (82/94). Data was collected from April-September 2013. Results for 82 participants were analyzed using SPSS version 21.

**Results:**

EPDS screened 35/82 (43 %) eligible antenatal attendees as depressed, out of which 16/35 (20 %) were severely depressed and 19/35 (23 %) were moderately depressed. Incidence of poor maternal dietary intake was more in females with antenatal depression. Antenatal depression increased the risk of poor Healthy Eating Index (RR = 2.58, C.I 1.60–5.23, AR = 62 %), carbohydrate <175 gm (RR = 1.188, CI 0.836–1.688, AR = 15 %), protein <71 gm (RR = 1.343, CI 1.059–1.703, AR = 26 %) and fat <55 gm (RR = 2.954, CI 1.612–5.416, AR = 67 %) Incidence of neonatal outcomes included, Fetal Growth Retardation (RR = 2.70, C.I 0.69–3.70, AR = 60 %), preterm birth (RR = 1.60, C.I 0.72–2.45, AR = 54 %), low Apgar score (RR = 2.70, C.I 0.69–3.70, AR = 60 %) and Low Birth Weight (RR = 0.56, C.I 0.93–1.39, AR = −44 %).

**Conclusion:**

Antenatal women with depression developed poor dietary intake and had increased incidence of Fetal Growth Retardation, preterm birth and low Apgar score, but not of Low Birth Weight.

## Background

Depression is one of highly prevalent mental disorders posing high disease burden. It is 50 % higher for females than males [1]. 10–15 % of women in developed countries and 20–40 % of women in developing countries experience depression during pregnancy or after childbirth [2]. Antenatal depression could lead to many health implications if not diagnosed and treated in time. It is a well-known fact that maternal morbidity and mortality not only affects families but also disrupts the social fabric of our communities and societies [3].

During pregnancy nutritional requirements are increased, to meet the needs of the fetus and mother. Adequate intake of calories and nutrients during pregnancy are necessary to have healthy mother and child at the end of antenatal period [4]. During pregnancy, higher depressive symptoms are associated with decreased healthy nutrition and increased unhealthy nutrition [5]. Depressed women tend to consume fewer macronutrients, except for fat than their non depressed counterparts [6]. Whole dietary intake levels of pregnant women were found to be insufficient as compared to Recommended Nutrient Intake with the exception of phosphorus [7]. On the contrary it was also found in literature that stressed pregnant women have increase in macronutrient intake, while decrease in some micronutrients [8].

Mental health of women adversely affects the nutritional intake as well as it impacts the developing fetus [9]. Many studies found that neonates of depressed mothers are also at greater risk for being Low Birth Weight (LBW) [10, 11], small for gestational age [12] and preterm birth [13, 14]. LBW is one of the leading causes of neonatal morbidity and mortality [15]. Neonates of depressed mothers have lower mean Apgar scores at 1 and 5 min after birth [12, 16]. While most studies support the inverse relationship between antenatal depression and neonatal outcomes, the results of others do not concur, suggesting that the evidence is still inconclusive [13, 17, 18].

In Pakistan maternal mortality rate is 260 deaths/100,000 live births [19] and data for physical, mental and psychological morbidity in antenatal period is insufficient to tell the exact burden. In Pakistan, incidence of antenatal depression ranges from 18 to 80 %, [16, 20–24], which goes undiagnosed, resulting in implications. There is definite need for investigation into the effects of antenatal depression on maternal dietary intake and neonatal outcome in our population. As improving maternal health is always a national and international priority [25] so there is a need to focus research on mental and psychological morbidities in Pakistan. Scarce literature is available on prevalence and incidence of antenatal depression and its effects on neonatal outcome in Pakistan but the effect on dietary intake of pregnant women is still an area which needs to be explored. Current study is aimed at measuring the association of depression with maternal dietary intake and neonatal outcome.

## Methods and materials

### Study design and settings

This was a hospital-based, closed, specific exposure cohort i.e. antenatal depression. Data collection took six months from April 2013 to September 2013. The study was conducted in a tertiary care, teaching hospital in the city of Lahore with a central geographic location, high antenatal out-patient turnover and good reputation for maternal and child health services in the town.

### Participants

Participants consisted of eligible antenatal attendees at the start of second trimester, aged 18–49 years, belonging to middle income group and having nutritional intake within normal range (using food frequency checklist and 24-h recall), so not to have one of the outcome of exposure present among cohort members at the beginning of the study. Antenatal attendees with history of known depression and chronic diseases (blood pressure, diabetes, severe anemia and CHD) or categorized as high risk pregnancy by a gynecologist were excluded from the study so solitary effects of depression could be observed.

Sample size was calculated using 95 % confidence and RR of 1.9 [11]. A sample size of 78 (39 in each cohort) was calculated. Assuming a 20 % non-response rate in cohorts, study was started with 94 antenatal women. All eligible antenatal attendees were consecutively enrolled in the study and were screened using Edinburgh Postnatal Depression Scale (EPDS) [26] to screen depressed as an exposure cohort and non-depressed antenatal attendees as internal comparison cohort, until the desired sample size of 47 in each group (94 in total) was completed. Out of these 94, 12 participants were lost to follow up, so analysis was carried out on a sample of 82, resulting in a dropout rate of 13 % among which 5 % were from exposed group.

### Instruments

Structured questionnaire was administered to collect data on potential confounders and effect modifiers including demography, anthropometry, parity, education, income level, husband’s employment, gestational age and expected delivery date. The questionnaire had been previously piloted for understandability and changes were made accordingly.

EPDS was used to screen antenatal depression. A score less than 9 indicated absence of depression, and a score of 9–12 indicated moderate depression and a score more than 13, severe depression. EPDS is a validated scale with Cronbach alpha 0.87 to screen antenatal and postnatal depression and has been previously used in Pakistani population [16, 24] and the standardized α coefficient is 0.87 [27]. The scale was translated in Urdu and re-translated in English 2 times before administration [28].

Middle class was determined after conversion and adjustment of Purchasing Power Parity [29, 30]. Middle class was taken as a family of 5, earning $ 123–504/month. The cohort was followed for incidence of poor maternal dietary intake and poor neonatal outcome till discharge after delivery.

### Assessment of maternal dietary intake

Maternal dietary intake was measured through 24 Hour Recall and Food Frequency Checklist at the start of cohort. Twenty-four hours recall and food frequency checklist was filled again in 36^th^ week of gestation to measure the incidence of poor maternal dietary intake. The Food Frequency Checklist assessed habitual diet by asking about the frequency with which food items were consumed over one week. Categories ranged from never to 6+ in a week. The food frequency checklist was modified in cultural context; foods not typically used in Pakistani diet were omitted and foods commonly used were added.

A 24 Hour Dietary Recall is a retrospective method of dietary assessment; the antenatal women were interviewed about types and amount of food and beverage consumption during the previous 24 h. The women were probed for portion sizes, method of preparation and snacks. Twenty-four hours intake was used to calculate the caloric intake, macronutrient i.e. carbohydrate, protein and fat content (in grams) by using Food Exchange lists.

The Healthy Eating Index (HEI) was used to analyze 24 h recall. HEI is a measure of diet quality and is a scoring metric that can be applied to any defined set of foods, such as formerly collected dietary data, to estimate a score [31]. A modified HEI was used, and the overall score was reduced to 50. Dietary intake was analyzed for adequacy components only; i.e. total fruit (5 score), whole fruit (5 score), total vegetables (5 score), greens & beans (5 score), whole grain (10 score), dairy (10 score), total protein foods (5 score) and seafood & plant proteins (5 score) only. Recommended intake was given full score and lesser were scored proportionately. A perfect adherence to dietary guidelines yielded a score of 50, a score ≥ 40 indicated good diet, score between 25–40 was rated as moderate and below 25 was considered poor diet.

To categorize carbohydrate intake a cut-off point of ≥ 175 g was used and for protein ≥ 71 g was used based on Recommended Dietary Allowances for pregnant women [32]. No RDA was available for fats, so minimum requirement for fat (20 %) was used to calculate minimum fat requirement from mean caloric requirement of the current sample (2500 kcal). Thus ≥ 55 g was used to categorize fat intake.

### Maternal anthropometric measurements

Height was measured using standard stadiometer to nearest 0.1 cm wearing light clothing without shoes. Weight was measured on a calibrated weighing scale and documented to nearest 100 g. The BMI of participants were calculated as weight (kg)/height (m)^2^. Participants were classified as underweight, normal, over weight and obese according to WHO criteria for body weight determination [33].

### Assessment of neonatal outcomes

After delivery, data on neonatal outcomes was obtained from patient files in hospital. Information was obtained about presence or absence of poor neonatal outcome taken as presence of FGR, preterm birth, low Apgar score and LBW among neonates before discharging from hospital. Fetal Growth Retardation (FGR) refers to poor growth of a baby while in the mother’s womb during pregnancy [34]. FGR was identified through ultrasonography reports evaluated by a radiologist or a gynecologist. LBW is defined as birth weight of a live born neonate of less than 2500 g [35] so a birth weight less than 2500 g was identified as LBW. A gestational age less than 36 weeks and 6 days is regarded as preterm birth [36] so neonates born before 36 weeks and 6 days were identified as preterm birth. Apgar is a system of assessing the general physical condition of a newborn. A score of 6 or less is rated as poor [37] so neonates with a score less than 6 were rated as poor Apgar. Apgar score had been determined by a trained neonatal nurse.

### Ethical considerations

Ethical approval for the study was obtained from the Institutional Review Board of Fatima Memorial Hospital, College of Medicine & Dentistry, Lahore, Pakistan. The research was conducted in compliance with the ethical principles for medical research involving human subjects of the Helsinki Declaration [38]. Written informed consent was obtained from all participants. The right to privacy, anonymity, voluntary participation and confidentiality were observed. Depressed women identified were provided with necessary information about their condition and where to seek medical advice.

### Statistical analysis

Demographic profile of the participants was developed, which revealed the percentage, means and standard deviation of the sample. Descriptive statistics and correlation of all the quantitative study variables were calculated. All the categorical variables such as baseline and end of cohort maternal dietary intake rating, FGR, preterm birth, LBW and low Apgar score, were analyzed in exposure and comparison cohorts. Relative risk and Attributable risk of antenatal depression on outcome variables was calculated. A probability level of *p* < 0.05 was taken as significant. Statistical analysis was carried out using Statistical Package for the Social Sciences (IBM SPSS Statistics for Windows, Version 21.0) [39].

## Results

Analysis of data on socio demographic variables of antenatal women revealed that out of 82 participants, 51 % were between the ages of 24–29 years. Mean age was 27 ± 4.4 years. 70 % of participants had received education above intermediate. The height of 66 % women ranged from 151–160 cm. The mean weight of mothers was 70 kg and mean BMI was 26.6. 41.5 % of respondents were primi-gravida (first conception) while average parity was 2.4 per woman. 69.5 % had no miscarriage or abortion history. 58 % of the women belonged to the family income group of $ 360–550/month and 30 % with 5 dependent family members. Husbands of 30 % of the antenatal women had their own business, 35 % worked in a private firm and 27 % were skilled laborers. 61 % reported having servants and 85 % were living as nuclear family (Table 1).

**Table 1:** Anthropometric and Socio-demographic characteristics of the mother

Characteristics		Percent	Mean	S.D
Mother’s Age (year)	18–23	21.9	26.9	4.4
	24–29	51.2		
	30–35	23.2		
	35+	3.7		
Mother’s Education	Uneducated	2.4	12.4	3.09
	Grade 5	1.2		
	Grade 8	3.7		
	Grade 10	22		
	Grade 12	23.2		
	Graduate	29.3		
	Post- Graduate	18.2		
Mother’s Height (cm)	141–150	12.2	157.1	4.9
	151–160	65.9		
	161–170	21.9		
Mother’s Weight (kg)	<54	7.3	70.4	12.1
	55–70	45.1		
	71–85	39.1		
	86–100	7.3		
	>100	1.2		
BMI	Normal	37.8	26.6	4.09
	Over-weight	46.4		
	Class I Obese	12.2		
	Class II Obese	2.4		
	Class III Obese	1.2		
Parity	1	41.5	2.4	1.38
	2	13.4		
	3	21.9		
	4	15.9		
	5	6.1		
	6	1.2		
Abortion/Miscarriage	0	69.5	0.5	0.86
	1	19.5		
	2	6.1		
	3	3.7		
	4	1.2		
Dependent Family Members	2	20	4.3	1.9
	3	17		
	4	16		
	5	30		
	6+	17		
Husband’s Occupation	Business	30		
	Private firm	35		
	Skilled labourer	27		
	Govt. job	2.4		
	Academia	2.4		
	Landowner	2.4		
Household Income ($)	160–250	26.8	372	130.3
	260–350	14.6		
	360–450	28.1		
	460–550	30.5		
Family System	Separate	85		
	Joint	15		
Servants	Yes	61		
	No	39		

Results of EPDS showed 35/82 (43 %) of participants were depressed (cut off point EPDS score 9). Out of these 16/35 (20 %) were severely depressed (EPDS score ≥13) and 19/35 (23 %) were moderately depressed (EPDS score 9–12).

Mean and standard deviation were calculated for both cohorts. Analysis of the characteristics of depressed antenatal women revealed in the baseline data rendered the mean age (26.8 years, ±4.3 S.D.), mean weight (70.6 kg, ±14.1 S.D.), mean BMI (26.6, ±4.7 S.D.), mean education (12.06 years, ±3.8 S.D.) and mean parity (2.2, ±1.4 S.D). These values did not significantly differ in the comparison cohort, demonstrating that characteristic of mother in both groups were identical at the start of the cohort. But mean household earning of depressed women was $ 37.51 lower than non-depressed women (Table 2).

**Table 2:** Relationship of maternal socio-demographic factors, dietary intake & neonatal outcomes with EPDS score

Variables of mother	Depressed antenatal women n = 35	Non-depressed antenatal women n = 47	EPDS score n = 82
	Mean (S.D)	Mean (S.D)	r (p-value)
Age (years)	26.8 (4.3)	26.8 (4.4)	−0.025 (0.824)
Weight (kg)	70.6 (14.1)	70.1 (10.5)	−0.107 (0.339)
BMI	26.6 (4.7)	26.6 (3.6)	−0.124 (0.267)
Household income ($)	350.57 (130.2)	388.08 (129.3)	−0.069 (0.536)
Education (years)	12.06 (3.8)	12.6 (2.5)	−0.032 (0.774)
Parity	2.2 (1.4)	2.4 (1.3)	−0.096 (0.390)
Baseline maternal dietary intake			
HEI 1	24.3 (6.6)	25.3 (6.6)	−0.103 (0.359)
Caloric intake 1	1344 (324)	1495 (322)	−0.290 (0.008)
Carbohydrate (gm)	173.9 (46.0)	167.8 (52.2)	−0.061 (0.585)
Protein (gm)	62.5 (22.2)	57.6 (18.0)	−0.121 (0.277)
Fat (gm)	54.1 (22.7)	69.4 (24.7)	0.306 (0.005)
End of cohort maternal dietary intake			
HEI 2	21.2 (5.3)	26.2 (5.0)	−0.406 (0.000)
Caloric intake 2	1219 (267)	1540 (280)	−0.524 (0.000)
Carbohydrate (gm)	161.5 (53.7)	166.5 (48.1)	0.049 (0.660)
Protein (gm)	57.0 (23.3)	60.0 (15.3)	0.078 (0.486)
Fat (gm)	50.4 (20.9)	72.6 (21.6)	0.462 (0.000)
Variables of neonate (end of cohort)			
Gestational age	38.1 (1.6)	38.3 (1.3)	0.065 (0.563)
Weight of neonate	2.7 (0.8)	2.9 (0.63)	0.084 (0.452)
Apgar score 0 min	7.1 (1.9)	7.4 (0.9)	0.120 (0.282)
Apgar score 5 min	8.3 (2.1)	8.8 (0.62)	0.182 (0.103)

Baseline maternal dietary intake (24 h recall) revealed that there was a mean difference of just one HEI score between depressed (24.3 HEI score) and non- depressed (25.3 HEI score) antenatal women. The depressed antenatal women were consuming an average of 151 kcal lesser than non depressed antenatal women at the start. At the end of cohort, a remarkable difference was evident between means of HEI score of depressed (21.2 HEI score) and non depressed (26.2 HEI score) antenatal women, a mean difference of 5 HEI scores with SD ± 5.3. Caloric intake of depressed antenatal women was further reduced by end of cohort, as depressed antenatal women were consuming 321 kcal less than non depressed antenatal women (Table 2).

A correlation coefficient for variables with EPDS score was calculated. At the baseline age, weight, BMI of the mother, parity, education, household income, HEI 1, carbohydrate and protein intake did not showed a correlation with EPDS score in both depressed and non depressed antenatal women. Yet the baseline fat intake had a moderate correlation with EPDS score (r = 0.306, *p* < α) which became stronger at end of cohort (*r* = 0.462, *p* < α). The baseline caloric intake showed a negative weak linear relationship with EPDS score (*r* = −0.290, *p* < α), which by the end of cohort increased to *r* = −0.524, *p* < α showing a moderate negative linear relationship. At the end of cohort, a moderate negative linear relationship between HEI 2 and EPDS score (*r* = −0.406, *p* < α) was observed (Table 2).

Food frequency checklist highlighted that the amounts of food consumed by the depressed and non depressed antenatal women were more or less the same for all food groups in the baseline dietary intake data. Cereals constituted the major proportion of the diet and its consumption was not affected by antenatal depression. The usage of eggs declined in the depressed group, by the end of cohort. Initially 43 % of depressed antenatal women never consumed eggs, which later increased to 75 %. The intake of lentils and beans remained the same. 80 % of depressed antenatal women were initially drinking milk on a regular basis which later dropped to 65 %. The fruit and vegetable intake of depressed antenatal women in current study was seen to be low. In the baseline dietary intake, 60 % of depressed antenatal women were having one serving of fruit daily but only 37 % consumed at least one fruit every day by the end of cohort. No one was eating green leafy vegetables even once a week.

Relative risk calculation at the end of cohort revealed that incidence of poor maternal dietary intake was more among antenatal depressed females than comparison cohort (R.R = 2.582, CI 1.60–5.23). 62 % of poor maternal dietary intake could be attributed to exposure to antenatal depression. At the end of the cohort, incidence of protein intake less than 71 g was more among depressed antenatal women (RR = 1.343, CI 1.059–1.703), 26 % of which could be attributed to antenatal depression. At the end of the cohort, incidence of fat intake less than 55 g was more among depressed antenatal women (RR = 2.954, CI 1.612–5.416), 67 % of which could be attributed to antenatal depression (Table 3).

**Table 3:** Risk estimate and chi square analysis of maternal dietary intake, neonatal outcomes with antenatal depression

		Depression	Depression	R.R^a^	A.R^b^ %	95 % C. I	p-value^c^
		Yes (%) n = 35	No (%) n = 47				
Maternal Dietary intake							
Baseline Healthy Eating Index	Poor	51	45	1.151	12	0.707–1.928	0.351
	Moderate	49	55				
Carbohydrate	<175 gm	57	53	1.074	7	0.720–1.591	0.823
	≥175 gm	43	47				
Protein	<71 gm	71	70	1.017	1	0.769–1.346	1.000
	≥71 gm	29	30				
Fat	<55 gm	51	30	1.727	43	1.002–2.976	0.067
	≥55 gm	49	70				
End of cohort Healthy Eating Index	Poor	71	28	2.582	62	1.603–5.228	0.000
	Moderate	29	72				
Carbohydrate	<175 gm	66	55	1.188	15	0.836–1.688	0.236
	≥175 gm	34	45				
Protein	<71 gm	89	66	1.343	26	1.059–1.703	0.021
	≥71 gm	11	34				
Fat	<55 gm	63	21	2.954	67	1.612–5.416	0.000
	≥55 gm	37	79				
Neonatal outcomes							
Fetal Growth Retardation	Yes	6	2	2.686	60	0.688–3.702	0.390
	No	94	98				
Preterm birth	Yes	17	11	1.611	54	0.727–2.452	0.297
	No	83	89				
Low Birth Weight	Yes	14	25	0.56	−44	0.292–1.393	0.167
	No	86	75				
Poor Apgar score	Yes	6	2	2.686	60	0.688–3.702	0.390
	No	94	98				

^a^ Relative Risk

^b^ Attributable Risk

^c^ Chi square

At the end of cohort neonatal outcomes were evaluated. Mean gestational age, weight, Apgar score 0 and 5 min were lower among neonates of depressed antenatal women. Neonates of depressed women were born 2 days earlier (38.1 verses 38.3 weeks) and weighed 200 g less (2700 verses 2900 gm). Apgar 0 min was 0.3 score lower and APGAR 5 min was 0.5 score lower than neonates of non depressed antenatal women. But correlation was not significant (Table 2).

Preterm birth (<37 weeks) was measured and categorized through gestational age. LBW (<2.5 kg) was categorized through birth weight and a score less than 7 was categorized as poor Apgar score. Incidence of FGR (RR = 2.686), preterm birth (RR = 1.611) and poor Apgar score (RR = 2.686) was higher among neonates of depressed antenatal women but not LBW (RR = 0.56). 60 % of FGR and poor Apgar score and 54 % of preterm births could be attributed to antenatal depression. Incidence of LBW was reduced by half among neonates of depressed antenatal women (Table 3).

## Discussion

Antenatal depression is like an iceberg in our society, effecting large proportion of antenatal women. The study was aimed at measuring the effect of depression on maternal dietary intake and neonatal outcome. A score of 9 was used as cut off point and the rate of depression was found to be 43 % in an urban set up, middle class, which was almost similar to results found in Gilgit Baltistan [23] in 2011 (48 %) and in Lahore [16] in 2010 (42 %). Both used EPDS to identify antenatal depression. A much lower prevalence was found in Hyderabad [20] (18 %) in 2009 and Chitral [22] 33.8 % in 2012. The discrepancy could be attributed to geographical differences and the measurement scales (AKUADS-a scale for measurement of anxiety and depression). Similar lower result was found in Rawalpindi [11] (25 %) in 2007 in which ICD-10 was applied; which is measurement of mental and behavioral disorders. Both AKUADS and ICD-10 are not specific for antenatal depression. Higher prevalence of depression was found in 2013 in Lahore [24] (80 %). The particularly high prevalence of antenatal depression found in the current study makes it a major public health problem.

To ensure internal validity, a control group was used, subjects were assigned to control and exposure group after screening with EPDS (valid and reliable tool for screening antenatal depression), pre tested to observe initial differences, study started well before development of outcome (poor neonatal outcome) and outcomes were measured and documented by a trained neonatal nurse. Data on potential confounders revealed that mean age, weight, BMI and parity of depressed antenatal women did not vary much from non depressed group. There was no relationship between variables of antenatal women and EPDS score. Half the participants were aged 24–29 and literacy level was quite good in both groups, only 2.5 % illiterate. Majority belonged to middle income level and husband’s of antenatal women mainly had their own business or worked in private firms. More than half reported having servants and majority lived as nuclear families or in separate units with their in-laws.

Antenatal depression affects not only mental health of the women but also has adverse effect on physical health by altered food habits and dietary intakes. A healthy and diverse diet is imperative at all times in life, but above all in pregnancy. Depressed women tend to consume fewer macronutrients, except for fat than their non depressed counterparts [6]. In the current study, the depressed antenatal women were consuming less of all macronutrients (carbohydrates, proteins and fats) than non depressed antenatal women at the end of cohort. Antenatal depression increased the risk of reduced protein and fat intake, which was statistically significant. The current study is in line with previous study reporting insufficient dietary intake levels of depressed pregnant women [7] but on the contrary to a study which reported an increase of macronutrients in depressed women [8].

Depression leads to poor nutrition and vice versa, giving rise to a whirlpool of increasing depression and decreasing nutrition [40]. Cross tabulation between depression and maternal dietary intake revealed that 51 % of depressed women had poor dietary intake which increased to 71 % by the end of the cohort. This shows the effect of depression on dietary intake of pregnant women, whereas 44 % non depressed antenatal women had poor dietary intake which decreased to 28 % by the end of the cohort. This change in dietary intake of non depressed women can be attributed to increased urge to eat to meet the maternal and fetal requirement in the last trimester. At the end of cohort, incidence of poor maternal dietary intake was associated with exposure to antenatal depression. The risk of having a poor maternal dietary intake was increased two and half times in depressed antenatal women. 62 % of poor maternal dietary intake could be attributed to exposure to antenatal depression. Statistical analysis was also significant *p* < α. These results were supports previous studies who found an inverse association between mean daily energy and antenatal depression [6] but not with Hurley’s study who found increased intakes of breads, fats, oils, sweets and snack group among depressed antenatal women [8].

Very few antenatal women had nutritionally adequate meal in a day as measured through 24-h recall and food frequency checklist. The depressed antenatal women had a reduced intake of milk, fruit and meat group. The iron intake of the antenatal women was considerably low as women’s diet was deficient in heme and non-heme sources. Vitamin B12 is only found in animal dietary sources and folic acid is found in fruits and vegetables. Low intake of meat group, fruits and vegetables may not only affect health of mother and the baby but may further precipitate maternal depression by depletion of nutrient reserves throughout the pregnancy [40].

Antenatal depression is also associated with adverse fetal and neonatal outcomes which include FGR, LBW, preterm birth, low Apgar score, neonatal morbidity and mortality. In the current study, incidence of FGR was found to be 2.686 times higher among depressed antenatal women than compared worth non depressed antenatal women (AR = 60 %). The results are in line with previous studies that high level of depression was associated with growth restriction in neonates [12, 41] except Andersson, who reported that in healthy populations antenatal depression was protective for FGR [17]. Incidence of preterm birth was 1.611 times higher among depressed antenatal women as compared to non depressed antenatal women (AR = 54 %). Similar results were found in other studies i.e. depressed women had greater incidence of premature delivery [13, 14, 41], but Goedhart and Andersson found no such association [12, 17]. Neonates of mothers with antenatal depression have low mean Apgar scores at 1 and 5 min. Incidence of poor Apgar score was 2.686 times more among depressed antenatal women as compared to non depressed antenatal women (AR = 60 %). The study supports the results of other researches [10, 12, 16].

Depression was found to be a protective factor against LBW (RR = 0.56, AR = −44 %). This could be because in our sample women belonged to middle SES, were receiving best antenatal care and patient compliance was very high regarding use of supplements. A positive and significant correlation between EPDS score and BMI of neonates had been previously observed [18], but Ibanez did not found an effect of antenatal depression on birth weight [13]. Other studies found negative effect of antenatal depression on weight of neonates [10, 17]. Depressed antenatal women had 15 % greater incidence of LBW [41]. In the current study, neonates of depressed antenatal women had a low birth weight (mean 2700 gm) than neonates of non depressed mothers (mean 2900 gm), which is similar to a study in Rawalpindi, Pakistan [11]. Although there was an increase in risk of FGR, low Apgar score and preterm birth associated with antenatal depression, but it was not found to be statistically significant.

The current study contributes in many ways, firstly to the best of my knowledge, it is the pioneer study conducted in Pakistan incorporating these variables. A few cross sectional surveys have been conducted for the screening of depression and its associated risk factors and only two focused on effects of depression on neonatal outcomes but none measured the effect of antenatal depression on maternal dietary intake, so, it is a rather unexplored sector in Pakistan. Secondly, the study contributes by being a prospective analytical cohort. Currently numerous birth cohorts are being conducted in developed countries but a national level research in Pakistan is lacking. Thirdly, the study population belonged to middle class which constitutes 55 % of Pakistan’s population [42]. Therefore, the study has updated the academia, especially in context to Pakistan’s urban middle class community.

## Conclusion

This study extends knowledge regarding the link between antenatal depression and maternal dietary intake and neonatal outcomes. This study has great implications for the mother, neonate and the healthcare provider. Ordinary health workers can identify depression with relative ease, which can help identify groups of mothers whose infants are at a greater risk, thus help in reducing neonatal morbidity. The effect of antenatal depression on dietary intake should be discussed during nutrition counseling in pregnant women.

## Abbreviations

AKUADS, The Aga Khan University Anxiety and Depression Scale; APGAR, appearance, pulse, grimace, activity, respiration; AR, attributable risk; BMI, body mass index; CI, confidence interval; EPDS, Edinburgh Postnatal Depression Scale; FGR, Fetal Growth Retardation; HEI, Healthy Eating Index; ICD-10, International Classification of Diseases; LBW, low birth weight; RR, relative risk

## References

[1] Mathers C, Fat DM, Boerma J. The global burden of disease: 2004 update. Geneva: World Health Organization; 2008.

[2] World Health Organization (2009). Mental health aspects of women’s reproductive health, a global review of literature.

[3] Mosley WH, Koblinsky MA, Reed HE. The Consequences of Maternal Morbidity and Maternal Mortality: Report of a Workshop. Washington, DC: National Academies Press; 2000. 25077262

[4] Mridlula D, Mishra CP, Ravorty C (2003). Dietary intake of expectant mother. Indian J Nutr Diet.

[5] Barker ED, Kirkham N, Ng J, Jensen SK (2013). Prenatal maternal depression symptoms and nutrition, and child cognitive function. Br J Psychiatr.

[6] Payab M, Motlagh A-rD, Eshraghian M, Rostami R, Siassi F, Abbasi B (2012). The association between depression, socio-economic factors and dietary intake in mothers having primary school children living in Rey, South of Tehran, Iran. J Diabetes Metab Disord.

[7] Kim HW (2011). Associations of dietary intake levels with ante-natal depression in pregnant women. Korean J Women Health Nurs.

[8] Hurley KM, Caulfield LE, Sacco LM, Costigan KA, Dipietro JA (2005). Psychosocial influences in dietary patterns during pregnancy. J Am Diet Assoc.

[9] World Health Organization. Maternal mental health and child health and development in low and middle income countries. Report of the WHO meeting. Geneva: World Health Organization; 2008.

[10] Kim HW, Jung YY (2012). Effects of antenatal depression and antenatal characteristics of pregnant women on birth outcomes: a prospective cohort study. J Korean Acad Nurs.

[11] Rahman A, Bunn J, Lovel H, Creed F (2007). Association between antenatal depression and low birth weight in a developing country. Acta Psychiat Scand.

[12] Goedhart G, Snijders AC, Hesselink AE, van Poppel MN, Bonsel GJ, Vrijkotte TG (2010). Maternal depressive symptoms in relation to perinatal mortality and morbidity: results from a large multiethnic cohort study. Psychosom Med.

[13] Ibanez G, Charles M-A, Forhan A, Magnin G, Thiebaugeorges O, Kaminski M (2012). Depression and anxiety in women during pregnancy and neonatal outcome: data from the EDEN mother–child cohort. Early Hum Dev.

[14] Orr ST, James SA, Prince CB (2002). Maternal prenatal depressive symptoms and spontaneous preterm births among African-American women in Baltimore, Maryland. Am J Epidemiol.

[15] MacDorman MF, Kirmeyer SE, Wilson EC. Fetal and perinatal mortality, United States, 2006. National vital statistics reports: from the Centers for Disease Control and Prevention, National Center for Health Statistics, National Vital Statistics System. 2012;60(8):1-22. 24979970

[16] Imran N, Haider II (2010). Screening of antenatal depression in Pakistan: risk factors and effects on obstetric and neonatal outcomes. Asia‐Pac Psychiat.

[17] Andersson L, Sundström-Poromaa I, Wulff M, Åström M, Bixo M (2004). Neonatal outcome following maternal antenatal depression and anxiety: a population-based study. Am J Epidemiol.

[18] Bunevicius A, Cesnaite E, Kusminskas L, Mockute I, Bunevicius R (2007). Antenatal mental state and anthropometric characteristics of the neonates: I. Impact of symptoms of depression and anxiety. Biol Psychiatry Psychopharmacol.

[19] CIA. World fact book South Asia. Pakistan: Central Intelligence Agency; 2013. https://www.cia.gov/library/publications/the-world-factbook/geos/print/country/countrypdf_pk.pdf. Accessed 5 Oct 2014.

[20] Karmaliani R, Asad N, Bann CM, Moss N, Mcclure EM, Pasha O (2009). Prevalence of anxiety, depression and associated factors among pregnant women of Hyderabad, Pakistan. Int J Soc Psychiatr.

[21] Rahman A, Iqbal Z, Bunn J, Lovel H, Harrington R (2004). Impact of maternal depression on infant nutritional status and illness: a cohort study. Arch Gen Psychiat.

[22] Mir S, Karmaliani R, Hatcher J, Asad N, Sikander S. Prevalance and risk factors contributing to depression among pregnant women in district Chitral, Pakistan. J Pak Psych Soc. 2012;9(1):28-36.

[23] Shah SMA, Bowen A, Afridi I, Nowshad G, Muhajarine N. Prevalence of antenatal depression: comparison between Pakistani and Canadian women. J Pak Med Assoc. 2011;61(3):242-6. 21465937

[24] Humayun A, Haider I, Imran N, Iqbal H, Humayun N. Antenatal depression and its predictors in Lahore, Pakistan. East Mediterr Health J. 2013;19(4):327-32. 23882957

[25] Economic UNDo. The Millennium Development Goals Report 2008. New York: United Nations Publications; 2008.

[26] Cox JL, Holden JM, Sagovsky R (1987). Detection of postnatal depression. Development of the 10-item Edinburgh Postnatal Depression Scale. Br J Psychiatr.

[27] Mental Health America of Georgia. Companion guide to the EPDS. 2014. http://www.mhageorgia.org/wp-content/uploads/2014/09/Companion_Guide_EPDS.pdf. Accessed 7 June 2015.

[28] Department of Health, Government of Western Australia (2006). Edinburgh Postnatal Depression Scale (EPDS): Translated versions – validated.

[29] Yuan Z, Wan GH, Khor N (2011). The rise of the middle class in the People’s Republic of China: Asian Development Bank.

[30] World Bank (2014). PPP conversion factor (GDP) to market exchange rate ratio.

[31] USDA (2013). Healthy eating index.

[32] National Academies of Sciences. Dietary Reference Intakes series. Wahington, DC: National Academies Press; 2011.

[33] WHO expert consultation. Appropriate body-mass index for Asian populations and its implications for policy and intervention strategies. Lancet. 2004;363:157-63. 10.1016/S0140-6736(03)15268-3 14726171

[34] U.S. National Library of Medicine (2014). Intrauterine growth restriction.

[35] Wardlaw TM. Low Birth Weight: Country, regional and global estimates: UNICEF; 2004. National Institutes of Health. Premature infants; 2011. (http://www.nlm.nih.gov/medlineplus/ency/article/001562.htm, Accessed 20 June 2014).

[36] National Institutes of Health (2011). Premature infants.

[37] Finster M, Wood M (2005). The Apgar score has survived the test of time. Anesthesiology.

[38] Association WM (2008). Declaration of Helsinki. Ethical principles for medical research involving human subjects.

[39] IBM Corp (2012). IBM SPSS Statistics for Windows, Version 21.0.

[40] Leung BM, Kaplan BJ (2009). Perinatal depression: prevalence, risks, and the nutrition link—a review of the literature. J Am Diet Assoc.

[41] Diego MA, Field T, Hernandez-Reif M, Schanberg S, Kuhn C, Gonzalez-Quintero VH (2009). Prenatal depression restricts fetal growth. Early Hum Dev.

[42] Ghani JA. The emerging middle class in Pakistan: how it consumes, earns, and saves. International conference on marketing. 2014. http://iba.edu.pk/testibaicm2014/parallel_sessions/ConsumerBehaviorCulture/TheEmergingMiddleClassPakistan.pdf. Accessed 14 July 2015.

